# Functional bias of contractile control in mouse resistance arteries

**DOI:** 10.1038/s41598-024-75838-8

**Published:** 2024-10-22

**Authors:** Nadia Haghbin, David M. Richter, Sanjay Kharche, Michelle S. M. Kim, Donald G. Welsh

**Affiliations:** 1 Department of Physiology and Pharmacology, Robarts Research Institute, Schulich School of Medicine and Dentistry, University of Western Ontario, London, ON Canada; 2 Department of Medical Biophysics, Robarts Research Institute, Schulich School of Medicine and Dentistry, University of Western Ontario, London, ON Canada

**Keywords:** Functional bias, Agonist-induced constriction, G-protein coupled receptors, Protein kinase C, Rho-kinase., Cardiovascular biology, Blood flow, Circulation

## Abstract

Constrictor agonists set arterial tone through two coupling processes, one tied to (electromechanical), the other independent (pharmacomechanical) of, membrane potential (V_M_). This dual arrangement raises an intriguing question: is the contribution of each mechanism (1) fixed and proportionate, or (2) variable and functionally biased. Examination began in mouse mesenteric arteries with a vasomotor assessment to a classic G_q/11_ (phenylephrine) or G_q/11_/G_12/13_ (U46619) agonist, in the absence and presence of nifedipine, to separate among the two coupling mechanisms. Each constrictor elicited a concentration response curve that was attenuated and rightward shifted by nifedipine, findings consistent with functional bias. Electromechanical coupling preceded pharmacomechanical, the latter’s importance rising with agonist concentration. In this regard, ensuing contractile and phosphorylation (CPI-17 & MYPT1 (T-855 & T-697)) measures revealed phenylephrine-induced pharmacomechanical coupling was tied to protein kinase C (PKC) activity, while that enabled by U46619 to PKC and Rho-kinase. A complete switch to pharmacomechanical coupling arose when agonist superfusion was replaced by pipet application to a small portion of artery. This switch was predicted, a priori, by a computer model of electromechanical control and supported by additional measures of V_M_ and cytosolic Ca^2+^. We conclude that the coupling mechanisms driving agonist-induced constriction are variable and functionally biased, their relative importance set in accordance with agonist concentration and manner of application. These findings have important implications to hemodynamic control in health and disease, including hypertension and arterial vasospasm.

**Supplementary Information:**

The online version contains supplementary material available at 10.1038/s41598-024-75838-8.

## Introduction

**Fig. 1:**
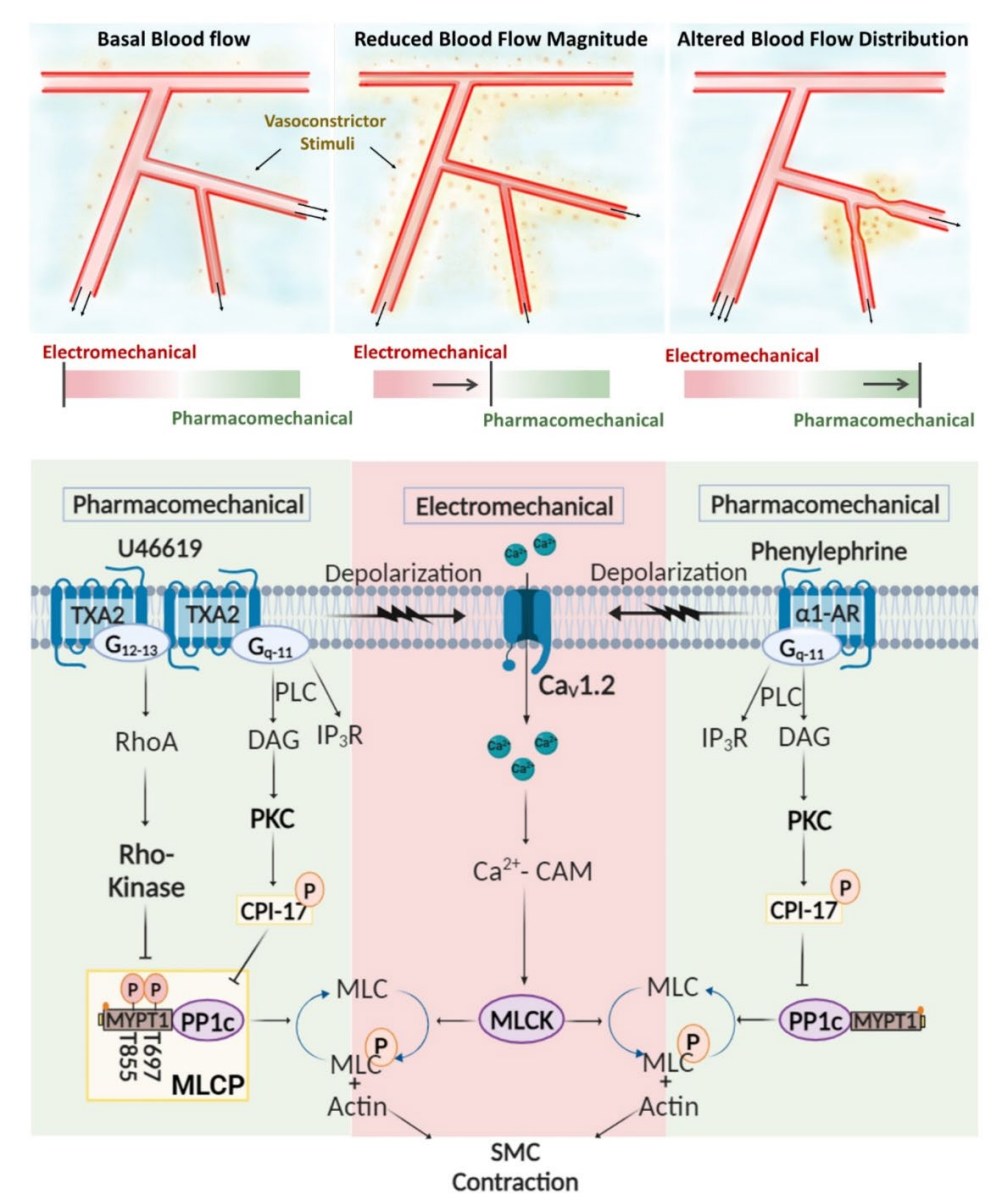
G protein-coupled receptors trigger electromechanical and pharmacomechanical coupling in a functionally biased manner to optimize blood flow delivery. Electromechanical coupling sequentially entails membrane depolarization, the activation of L-type Ca^2+^ channels, and a rise in cytosolic [Ca^2+^] that activates myosin light chain kinase (MLCK) to initiate cross-bridge cycling. Pharmacomechanical coupling regulates constriction in a voltage insensitive manner by inhibiting myosin light chain phosphatase (MLCP). This inhibition is enabled by Rho-kinase and CPI-17, protein kinases that target the catalytic (PP1c) and targeting (MYPT1) subunit of MLCP.

Resistance arteries form an integrated vascular network responsible for optimizing blood flow delivery to metabolically active cells^1,2^. In peripheral tissue, like skeletal muscle or the gut, basal tone is initially set by intravascular pressure and the release of neurotransmitters from sympathetic nerves. Each constrictor stimuli works through G-protein coupled receptors and associated transduction pathways to govern electromechanical and pharmacomechanical coupling, processes that set the phosphorylation state of the myosin light chain^3–5^. Electromechanical coupling is defined as the modulation of myosin light chain kinase (MLCK) through changes in membrane potential (V_M_) that drive an influx of Ca^2+^ through voltage-gated Ca^2+^ channels^6,7,8^. Pharmacomechanical coupling classically refers to the regulation of myosin light chain phosphatase (MLCP) through signal pathways that activate two key kinases, the first protein kinase C (PKC), the second Rho-kinase. The former targets CPI-17 (myosin phosphatase inhibitory protein, 17 kDa), a modulator of the MLCP catalytic subunit, PP1cδ, whereas the latter phosphorylates MYPT1, the targeting subunit of MLCP, via its T-697 and T-855 regulatory sites (Fig. 1)^9–11^.

Vascular studies have long recognized that constrictor agonists use both electro- and pharmacomechanical coupling to set arterial tone^12^. This knowledge raises a fundamental question: why do agonists engage two distinct coupling mechanisms, particularly in fixed relative proportion, irrespective of the agent presented or the receptors activated. Perhaps, one answer lies in recognizing the difficulties in optimizing blood flow magnitude and distribution in complex networks encoded by a single mechanism, for example electromechanical coupling^13–15^. A second mechanism with distinct contractile properties would thus be seemingly important, particular if recruited in a flexible manner relative to the first^16^. Variable contractile engagement could in theory arise from signaling bias intrinsic to G-protein coupled receptors and their associated downstream pathways^17^. The idea that the contractile mechanisms aren’t fixed in proportion to one another but are flexibly and “functionally biased” toward electro- or pharmacomechanical coupling is a novel concept worthy of interrogation. If validated through experimentation, this concept could profoundly alter our understanding of integrated blood flow control in physiological settings and with the genesis of vascular dysfunction.

This study determined whether functionally biased responses were observable in mesenteric arteries exposed to a G_q/11_ (phenylephrine) or G_q/11_/G_12/13_ (U46619) coupled receptor agonist, analogs akin to those released from perivascular nerves or endothelial cells/platelets, respectively. Our examination began by monitoring concentration response curves prior to and following pharmacological manipulation and subsequently to measurements of protein phosphorylation, membrane potential (V_M_), and cytosolic Ca^2+^ to garner deeper insights. We observed compelling evidence of functional bias, each agonist eliciting a contractile response where electromechanical coupling preceded pharmacomechanical, the latter increasing in prominence as concentrations were raised. The pharmacomechanical coupling response induced by phenylephrine was strongly linked to PKC, whereas that induced by U46619 was tied to both PKC and Rho-kinase activation. Ensuing experiments reveal how functional bias switches to complete pharmacomechanical dominance, when agent application is restricted to a small portion of the artery, a finding aligned with computational modeling. We conclude from this foundational work that constrictors elicit functionally biased responses and that arteries toggle between the contractile mechanisms. We discuss how this knowledge advances our mechanistic understanding of hemodynamic control in health and disease states, including arterial vasospasm.

## Results

### G-Protein coupling and the α-1 adrenergic and thromboxane A2 (TXA2) receptors

**Fig. 2:**
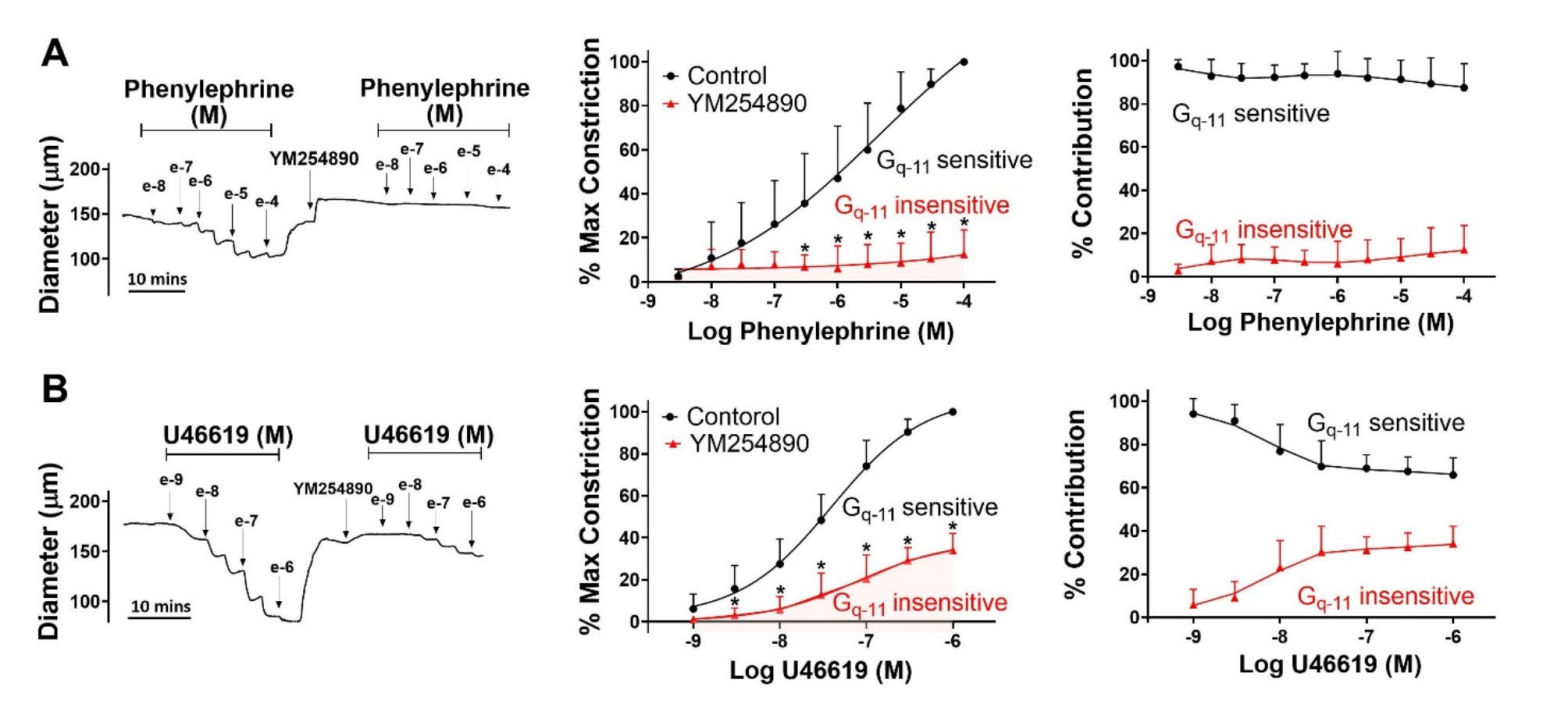
Effect of G_q−11_inhibitor on agonist induced constriction. Isolated mesenteric arteries from C57BL/6 mice were exposed to phenylephrine or U46619 in presence and absence of 0.1 µM YM254890 (G_q−11_ inhibitor). (**A**,** B**) Representative traces and summary data (% maximum constriction; % contribution of G_q−11_ sensitive and insensitive components) were calculated. P values for increasing phenylephrine concentration: 0.942, 0.658, 0.280, 0.0828, 0.0279, 0.0191, 0.00621, 0.000831, < 0.0001, < 0.0001. P values for increasing U46619 concentration: 0.12, 0.0226, 0.00043, < 0.0001, < 0.0001, < 0.0001, < 0.0001. (Two-way ANOVA, * indicates a P-value < 0.05). *n* = 6 arteries from 6 mice for each experiment. Data are mean ± SD.

To probe the nature of G-protein coupling, agonist-response curves to phenylephrine or U46619 were constructed in the absence and presence of a G_q/11_ inhibitor (YM254890)^18,19^. Figure 2 reveals that phenylephrine (3 × 10^− 9^ M to 10^− 4^ M) and U46619 (10^− 9^ M to 10^− 6^ M) induced robust concentration-dependent constrictions in mouse mesenteric arteries. Superfusion of YM254890 abolished phenylephrine-induced constriction (Fig. 2A) while its impacts on the U46619 response was mixed; a near complete elimination was observed when agonist concentration was low but only partial at higher concentrations (Fig. 2B). These findings align with α1-adrenoreceptors being singularly coupled to G_q/11_ while TXA_2_ receptors to both G_q/11_ and G_12/13_^34^.

## Sequential activation of contractile mechanisms

**Fig. 3:**
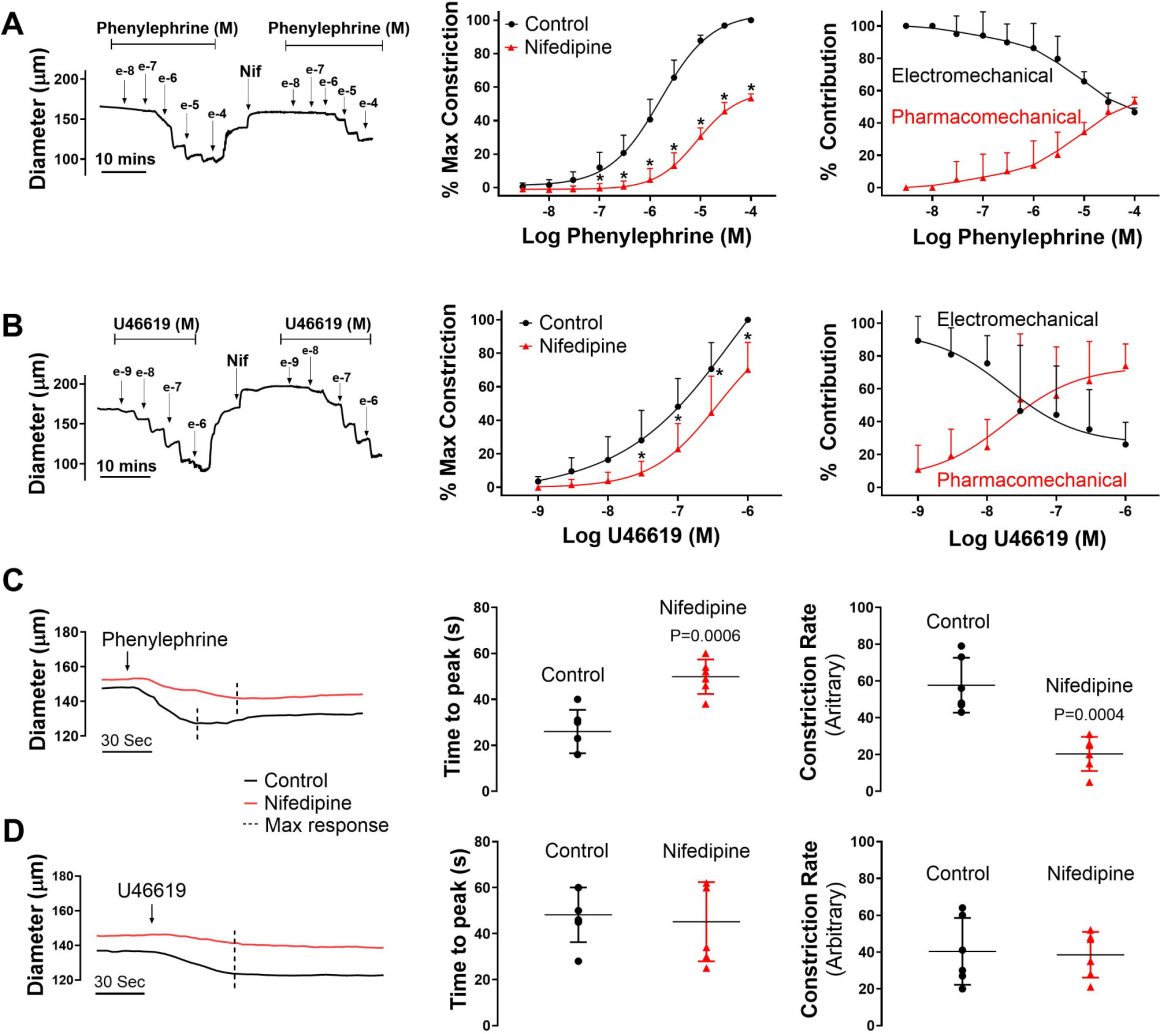
Relative contribution of electro- and pharmacomechanical coupling to agonist induced constriction. Isolated mesenteric arteries from C57BL/6 (wild type) mice were exposed to phenylephrine (**A**) or U46619 (**B**) in presence and absence of 0.3 µM nifedipine (L-type Ca^2+^ channel blocker). Representative traces and summary data (% maximum constriction; % contribution pharmacomechanical and electromechanical components) were calculated. P values for increasing phenylephrine concentration: >0.999, 0.995, 0.715, 0.007, < 0.0001, < 0.0001, < 0.0001, < 0.0001, < 0.0001, < 0.0001. P values for increasing U46619 concentration: 0.982, 0.480, 0.0764, 0.0012, < 0.0001, < 0.0001, < 0.0001. Time-to-peak constriction and constriction rate to 30 µM phenylephrine (**C**) and 0.3 µM U46619 (**D**) in isolated mouse mesenteric arteries. P value for phenylephrine time to peak constriction and constriction rate, respectively: 0.0006, 0.0004. P value for U46619 time to peak constriction and constriction rate, respectively: 0.671, 0.218. (Two-way ANOVA, paired *t*-test, * indicates a P-value < 0.05). *n* = 6 arteries from 6 mice for each experiment. Data are mean ± SD.

To address how electro- and pharmacomechanical coupling are engaged, agonist-response curves were constructed in the absence and presence of nifedipine, an L-type Ca^2+^ channel blocker that uncouples V_M_ from arterial constrictor control. Akin to the preceding work, the superfusion of phenylephrine (3 × 10^− 9^ M to 10^− 4^ M) or U46619 (10^− 9^ M to 10^− 6^ M) induced constriction of mouse mesenteric arteries in a concentration dependent manner (Figure Concentration response data points 3A & 3B, left and middle). The subsequent bath application of nifedipine diminished vessel reactivity and shifted the concentration-response curves to both agonists rightward. The nifedipine-sensitive component (electromechanical) dominated tone control at low agonist concentrations whereas the nifedipine-insensitive component (pharmacomechanical) rose to prominence at higher concentrations (Fig. 3A and B, right). Such findings are indicative of a functional bias in vascular reactivity, with electromechanical coupling preceding pharmacomechanical. Intriguingly, nifedipine’s impact on phenylephrine-induced constriction was more pronounced than on U46619, the latter transduced through the TXA2 receptor via G_q/11_ and G_12/13_ (Fig. 2A and B, right). Deeper analysis revealed that time-to-peak constriction (phenylephrine), was also moderated by nifedipine application, a finding in line with quicker temporal engagement of electromechanical coupling; a similar time shift wasn’t observed with U46619 (Fig. 3C and D). These time-dependent assessments are reported at agonist concentrations that elicited approximate 70–80% maximal constriction (10^− 5^ for phenylephrine vs. 3*10^− 7^ for U46619). Higher paired concentrations (3*10^− 6^ for phenylephrine vs. 3*10^− 8^ for U46619; 3*10 − 5 for phenylephrine and 10^− 7^ for U46619) yield qualitatively similar results (data not shown).

**Fig. 4:**
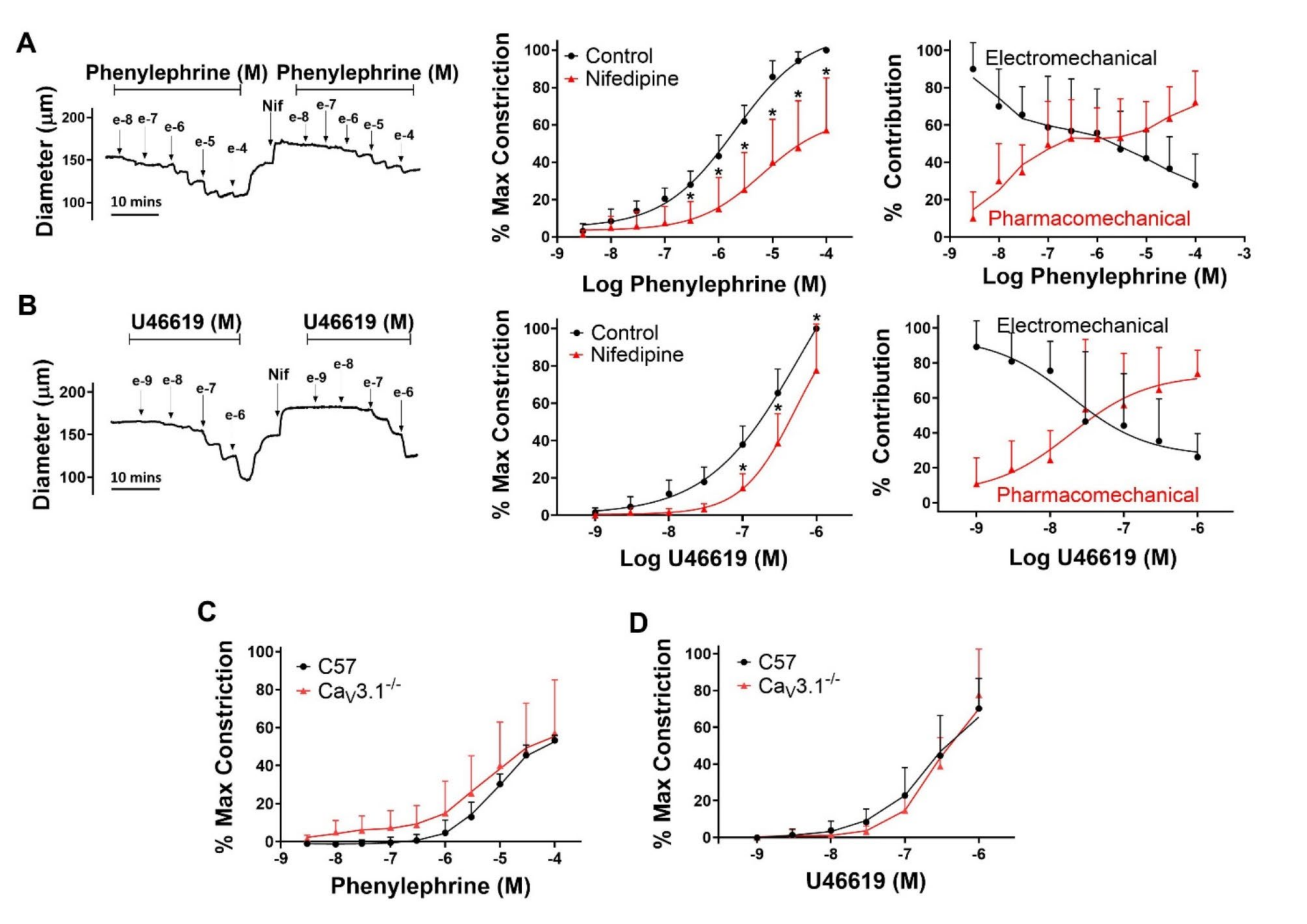
Contribution of T-type Ca^2+^channels in agonist induced constriction. Isolated mesenteric arteries from Ca_V_3.1^−/−^ mice were exposed to phenylephrine (**A**) or U46619 (**B**) in the presence and absence of 0.3 µM nifedipine (L-type Ca^2+^ channel blocker). Representative traces and summary data (% maximum constriction; % contribution pharmacomechanical and electromechanical components) were calculated. (**C & D**) Summary data compares the % of nifedipine insensitive constriction to phenylephrine and U46619 in arteries from wild type and Ca_V_3.1^−/−^ mice. P values for increasing phenylephrine concentration: >0.999, 0.999, 0.832, 0.254, 0.0169, 0.0001, < 0.0001, < 0.0001, < 0.0001, < 0.0001. P values for increasing U46619 concentration: >0.999, 0.998, 0.473, 0.0866, 0.0014, 0.0002, 0.0022. (Two-way ANOVA, * indicates a P-value < 0.05,). *n* = 6 arteries from 6 mice per group. Data are mean ± SD.

In recognition that T-type Ca_V_3.1 channels are expressed in vascular smooth muscle and could confound the interpretation of Fig. 3A and B, experiments were repeated in mesenteric arteries from Ca_V_3.1^−/−^ mice^20,21^. Agonist induced constriction (Fig. 4A and B, left and middle) in Ca_V_3.1^−/−^ arteries, appeared quantitatively similar to wild type control (Fig. 3A and B), in the absence and presence of nifedipine to block L-type Ca^2+^ channels. Each agonist increased tone in a concentration dependent manner, with nifedipine reducing and rightward shifting the constrictor response curves. Similar to wild type arteries, electromechanical coupling dominated at low agonist concentrations whereas pharmacomechanical coupling rose in prominence at higher concentrations (Fig. 4A and B, right). The head-to-head comparison (C57 vs. Ca_V_3.1^−/−^) in Fig. 4C and D, conducted with nifedipine to better visualize a T-type Ca^2+^ channel contribution revealed agonist-induced constriction was comparable among these groups.

## Pharmacomechanical coupling and phosphorylation control of MLCP

**Fig. 5:**
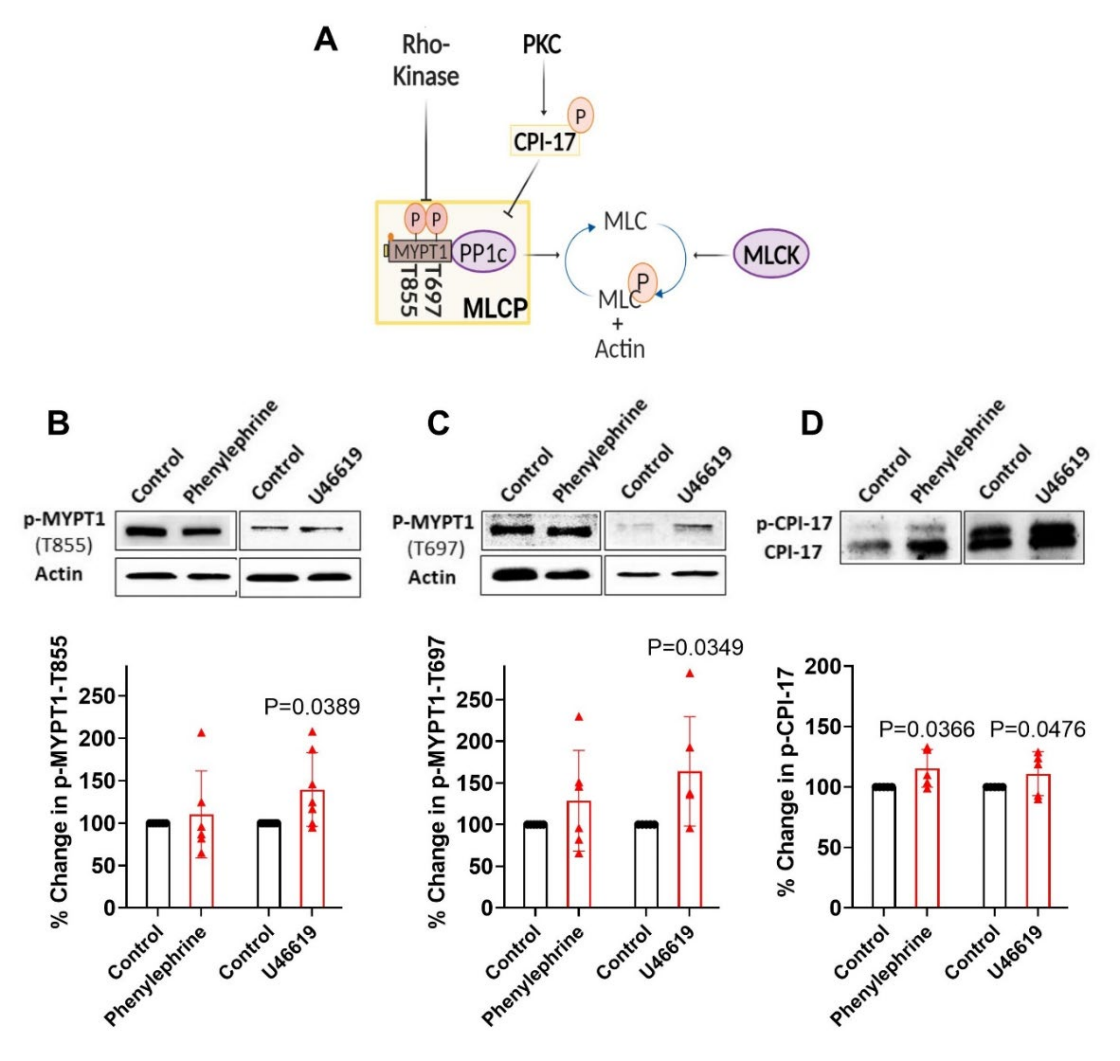
Agonist induced phosphorylation of MLCP regulatory targets. **A**) Key phosphorylation targets (MYPT1, T-855 & T-697 and CPI-17) in response to phenylephrine (30 µM) and U46619 (0.1 µM) application. Summary data shows Phenylephrine treatment increased the phosphorylation state of CPI-17 (**B**); in comparison U46619 treatment increased the phosphorylation state of CPI-17 (**B**) and MYPT1 (T-855 & T-697) **(C & D)**. α-smooth muscle actin was used as a loading control and % changes in p-MYPT1 was normalized to actin. Raw data was used for analysis. Original blots are presented in supplementary dataset file. The grouping of blots cropped from different parts of the same gel. P value in MYPT1-T855 in phenylephrine and U46619 group respectively: 0.894, 0.0389. P value for MYPT1-T697 in phenylephrine and U46619 group respectively: 0.4354, 0.0349. P value for CPI-17 in phenylephrine and U46619 group respectively: 0.0366, 0.0476 (Paired *t-*test) n = 5–7 samples from 5–7 mice for each experiment.)Data are mean ± SD.

**Fig. 6:**
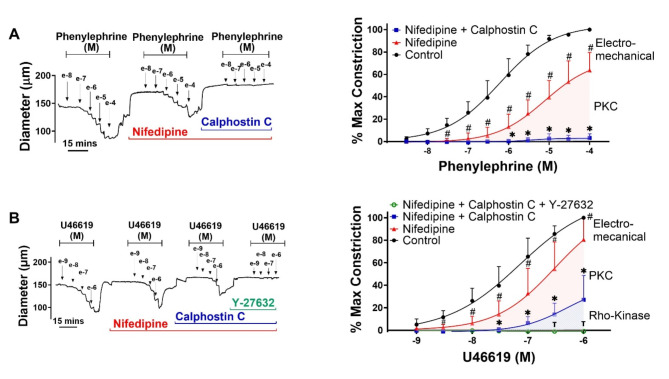
Effects of PKC and Rho-kinase inhibition on agonist induced pharmacomechanical coupling. (**A**) Representative trace and summary data of phenylephrine induced constriction with or without 0.3 µM nifedipine and 0.3 µM calphostin C. (**B**) Representative trace and summary data of U46619 induced constriction with and without 0.3 µM nifedipine, 0.3 µM calphostin C and 20 µM Y-27,632. P values for increasing phenylephrine concentration (#): 0.843, 0.207, 0.0021, < 0.0001, < 0.0001, < 0.0001, < 0.0001, < 0.0001, < 0.0001, < 0.0001. (*) > 0.999, 0.996, 0.9965, 0.707, 0.461, 0.023, < 0.0001, < 0.0001, < 0.0001, < 0.0001. P values for increasing U46619 concentration (#): 0.182, 0.013, 0.001, 0.0009, 0.0009, 0.0163, 0.0388. (*): 0.0965, 0.0746, 0.0353, 0.0296, 0.0256, 0.0075, 0.004. (Ʈ): 0.959, 0.578, 0.0973, 0.865, 0.0802, 0.0322, 0.0464. # denotes significant decrease from control curve. * denotes significant decrease from nifedipine-insensitive curve. Ʈ denotes significant decrease from PKC-insensitive curve. (Two-way AVOVA, P-value < 0.05 indicates significance). *n* = 6 arteries from 6 mice per group. Data are mean ± SD.

Pharmacomechanical coupling is intimately tied to MLCP regulation and the phosphorylation of three key sites one on CPI-17, an upstream modulator of PP1cδ, and two on MYPT1 (T-855 & T-697)^22,23^. We employed western blot analysis to examine these sites, prior to and following the application of agonists at concentrations that elicit near maximal constriction. Our findings reveal that a 3 min application of phenylephrine enhanced CPI-17 phosphorylation while having no significant effect on the phosphorylation state of MYPT1 (Fig. 5). U46619 application also enhanced CPI-17 phosphorylation, but in addition, markedly elevated MYPT1 T-855 & T-697 phosphorylation. CPI-17 phosphorylation is driven by PKC, and consistent with this kinase driving pharmacomechanical coupling, calphostin C (a pan-PKC inhibitor) fully and partially abolished the nifedipine-insensitive constriction by phenylephrine and U46619, respectively (Fig. 6)^11^. Complete elimination of U46619-induced pharmacomechanical response required the further inhibition of Y-27,632, a Rho-kinase inhibitor (Fig. 6B)^24,25^. These phosphorylation patterns highlight specific roles of PKC and Rho-kinase, the former (phenylephrine & U46619) mediating CPI-17 phosphorylation, and the latter (U46619) enhancing MYPT1 phosphorylation.

## Altering the order and dominance of coupling mechanisms

**Fig. 7:**
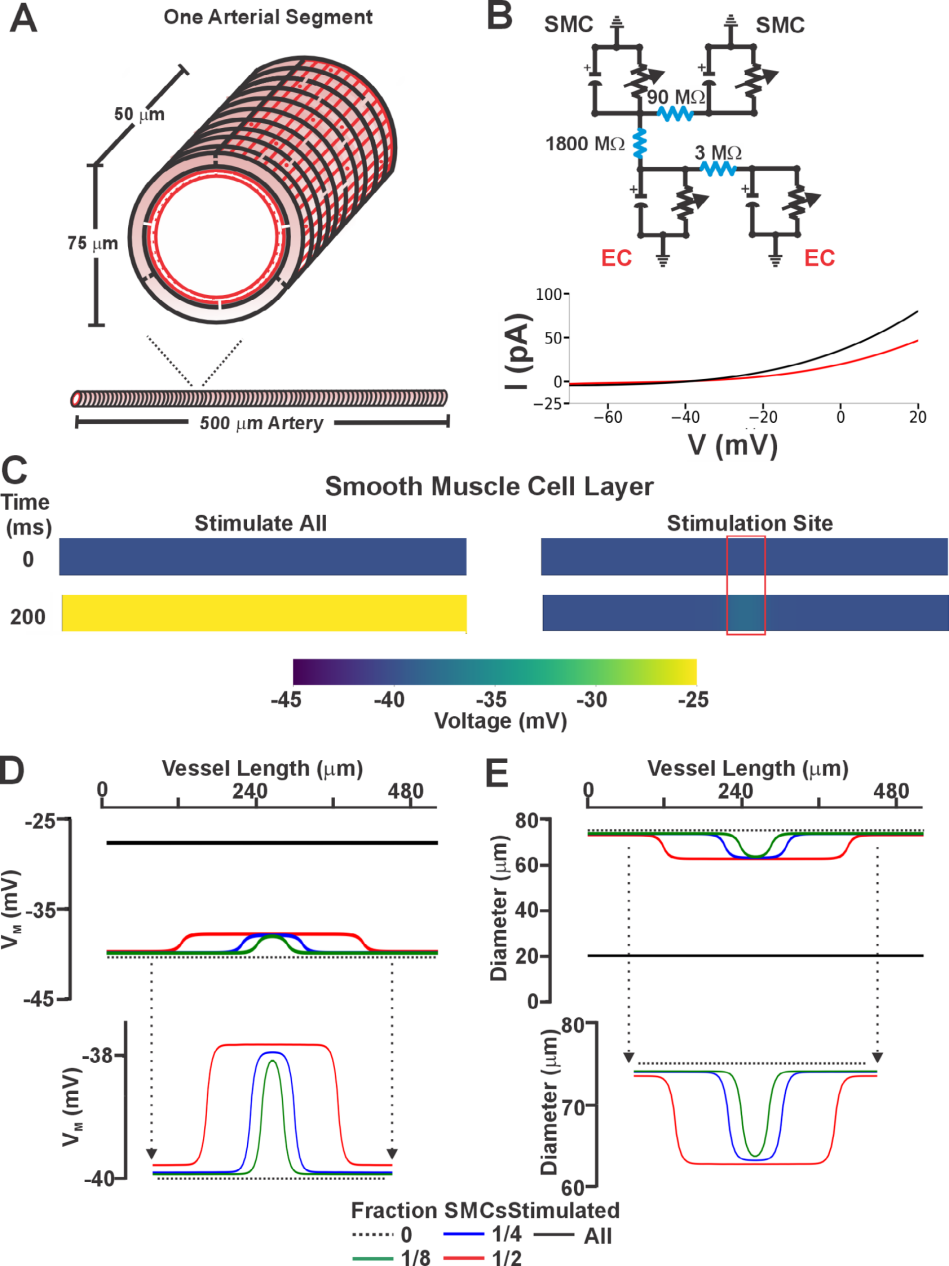
Virtual artery simulations: Smooth muscle cell depolarization in response to current injection. (**A**) The virtual artery of length 500 μm was composed of a single inner endothelial layer (depicted in red) and an outer smooth muscle layer (depicted in black). Each section of the artery consisted of 48 endothelial cells and 10 smooth muscle cells. Adjacent smooth muscle cells were electrically coupled as were neighboring endothelial cells; each smooth muscle cell was in turn coupled to two endothelial cells. (**B**) Each cell was modeled as a capacitor in parallel with a nonlinear resistor representing the ionic conductivity of the cell membrane. Gap junctions were represented as ohmic resistors. The current-voltage (I-V) characteristics of the nonlinear resistors are shown. (**C**) Voltage distributions of V_M_ as the whole artery (left) or 1/8th (right, red box) of all smooth muscle cells were injected with 0.83 pA of depolarizing current (200 ms). (**D & E)** Voltage and vasomotor outcomes for virtual arteries where a 0.83 pA stimulus was injected into all, or 1/2, 1/4 and 1/8 of all smooth muscle cells. Note the stimulation of all smooth muscle cells resulted in the virtual artery depolarized from − 40 to − 28 mV; stimulation of 1/8 of all smooth muscle cells reduced the electrical response to < 2.0 mV.

**Fig. 8:**
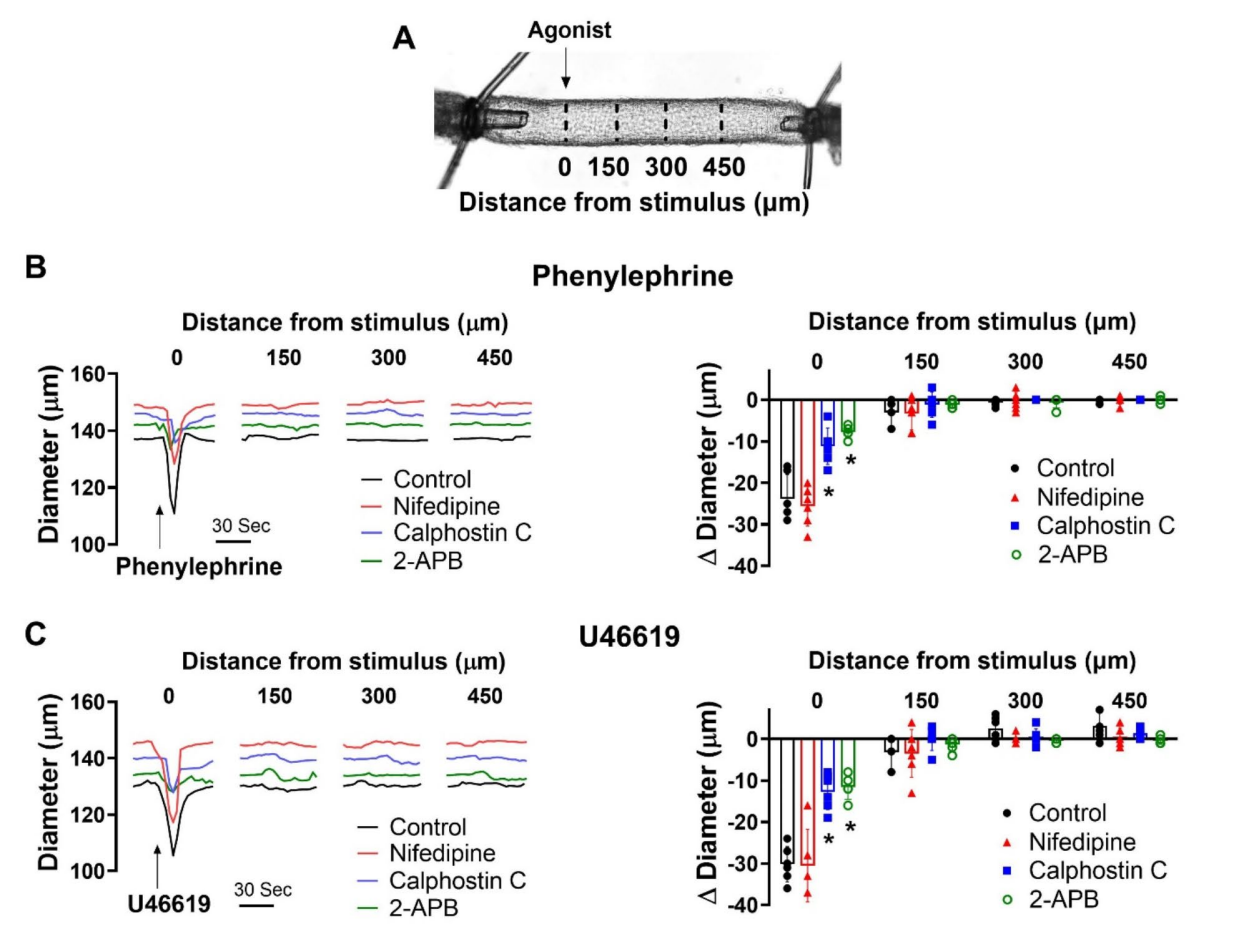
Effects of nifedipine and PKC inhibition on focal constriction. (**A**) Agonists were applied onto a small segment of mesenteric arteries using a glass micropipette; diameter was monitored 0–450 μm distal to the point of agent application. Representative traces and summary data of phenylephrine (**B**; 100 µM focally, 10 s pulse) or U46619 (**C**; 10 µM, 10 s pulse) application, with and without 0.3 µM nifedipine, 0.3 µM calphostin C or 50 µM 2-APB. * denotes significant decrease from control. (**B**) P value for Δ diameter at 0, control vs. nifedipine: 0.942, control vs. calphostin C: 0.0116, and control vs. 2-APB: 0.0198. (**C**) P value for Δ diameter at 0, control vs. nifedipine: 0.663, control vs. calphostin C: 0.0412 control vs. 2-APB: <0.0001. (Two-way ANOVA). *n* = 6 arteries from 6 mice for each experiment. Data are mean ± SD.

**Fig. 9:**
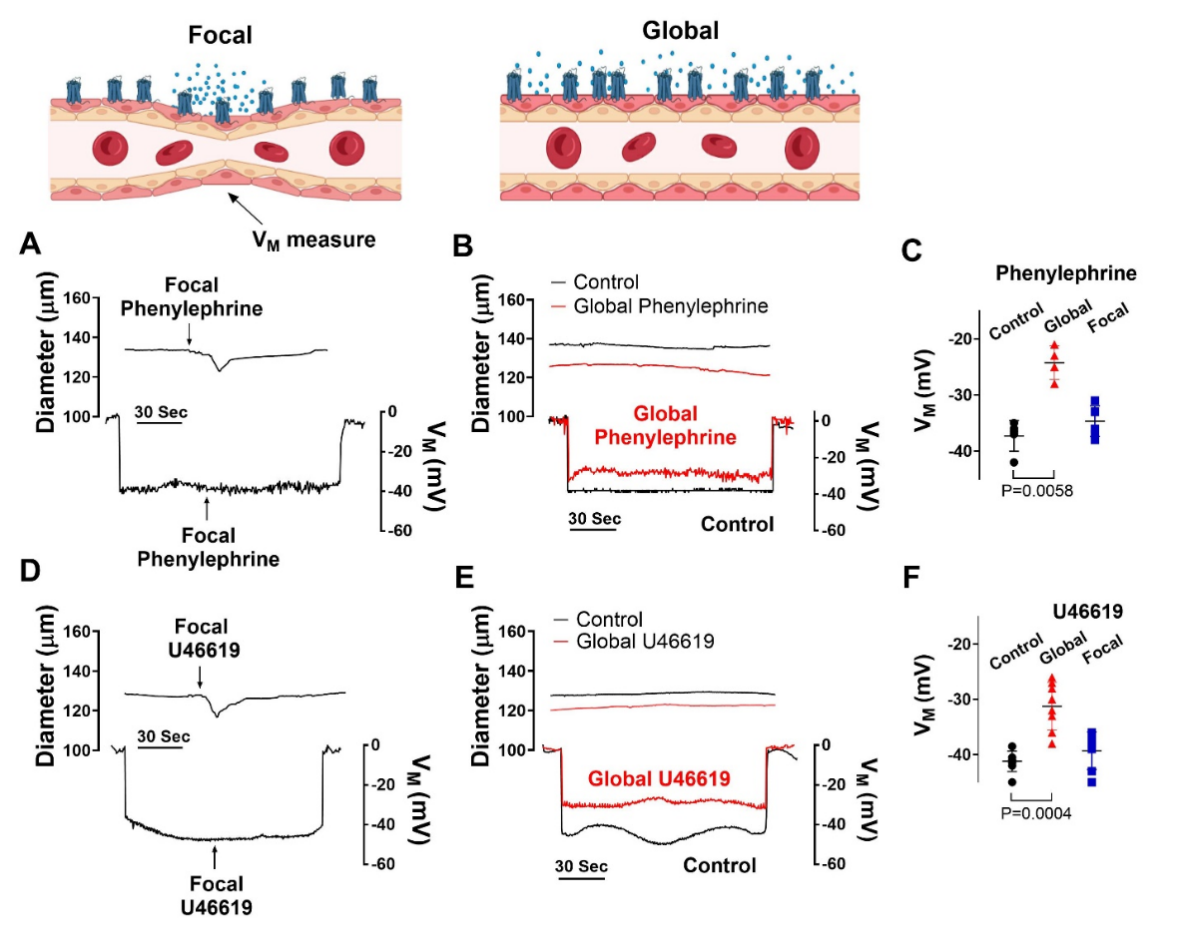
Focal agonist application does not induce arterial depolarization. Isolated mesenteric arteries from C57BL/6 mice were exposed to U46619 (10 µM focally, 10 s pulses or 0.1 µM globally) and phenylephrine (100 µM focally, 10 s pulses or 30 µM globally) was applied to mesenteric arteries while diameter and V_M_ were monitored. Representative tracing **(A & B)** and summary data **(C)** of responses to focal and global phenylephrine. Representative tracing **(D & E)** and summary data **(F)** of responses to focal and global U46619. P value for V_M_, control vs. global phenylephrine: 0.0058, control vs. focal phenylephrine: 0.367. P value for V_M_, control vs. global U46619: 0.0004, control vs. focal U46619: 0.1350. (Paired *t-*test). *n* = 6–8 arteries from 6–8 mice for each experiment. Data are mean ± SD.

**Fig. 10:**
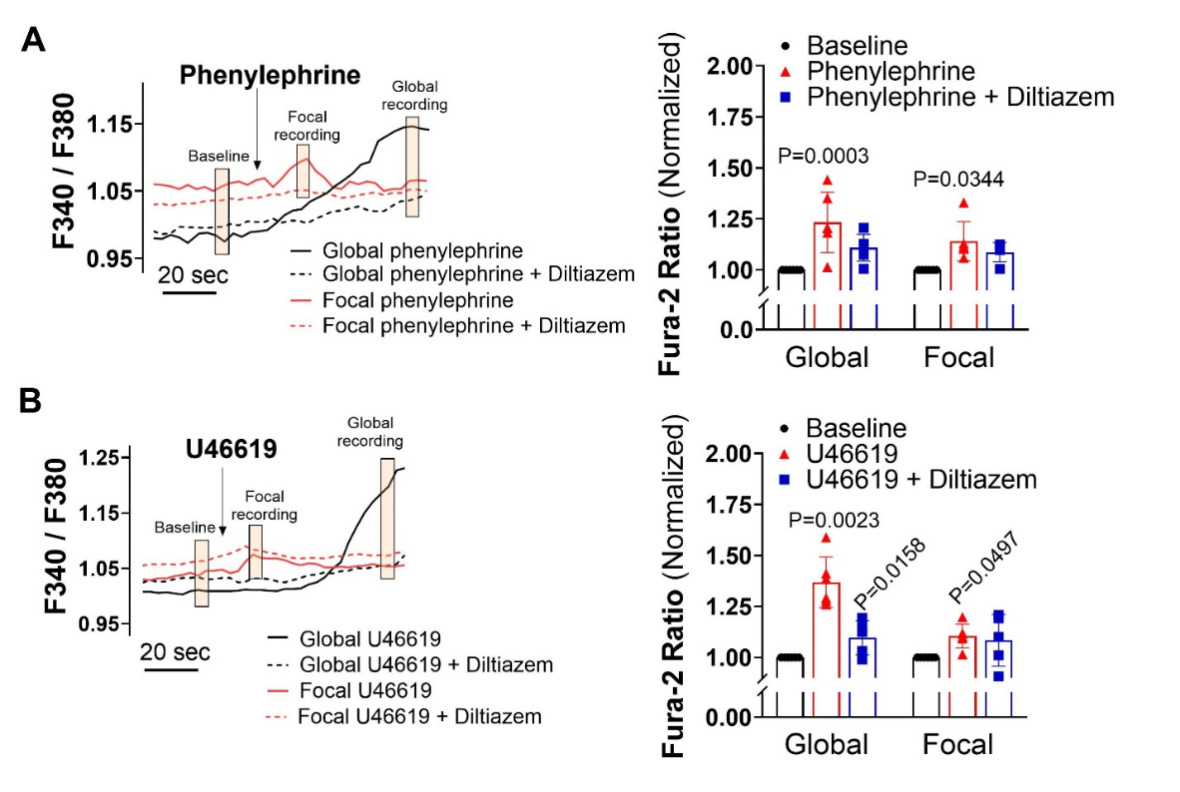
Effects of focal and global agonist application on intracellular [Ca^**2+**^]. Representative traces and summary data show the cytosolic Ca^2+^ changes (F340/F380) in response to focal and global phenylephrine **(A)** and U46619 **(B)** with and without diltiazem (30 μm, L-type channel blocker). Summative cytosolic Ca^2+^ data are normalized to baseline. **(A)** P values for Fura-2 ratio, baseline vs. global: 0.0003, global vs. diltiazem: 0.224, baseline vs. focal: 0.0344, focal vs. diltiazem: 0.988. **(B)** P values for Fura-2 ratio, baseline vs. global: 0.0023, global vs. diltiazem: 0.0158, baseline vs. focal: 0.0497, focal vs. diltiazem: 0.964 (Two-way ANOVA). *n* = 6 arteries from 6 mice for each group. Data are mean ± SD.

Based on the preceding work, we considered whether there existed a functional scenario where the order and dominance of coupling mechanisms is reversed. Modeling of electromechanical control reveals one possibility which centers on changing the number of smooth muscle cells generating a depolarizing current^28^. When all smooth muscle cells produce a small depolarizing current, we observed a + 12.5 mV response with corresponding constriction, a virtual result that aligns with our experiments in Fig. 7. That depolarization, however, decreased markedly to < 2 mV as the number of stimulated SMCs was reduced to 1/2 1/4 and 1/8 of total. With results implying pharmacomechanical coupling dominates when agonist presentation is restricted, we began testing this scenario by focally applying phenylephrine (100 µM) or U46619 (10 µM) via pipet onto a small portion of a mesenteric artery. Consistent with theory, discrete agonist application elicited a focal constriction resistant to nifedipine, a response indicative of its voltage insensitivity. Further contractile work indicated that pharmacomechanical coupling dominates as Calphostin C (PKC blocker) and 2-APB (IP_3_ receptor blocker) attenuated the focal constriction enabled by phenylephrine or U46619 (Fig. 8). Subsequent V_M_ measurements align with the vasomotor observations, with superfused agonist (phenylephrine or U46619) eliciting a profound arterial depolarization, whereas focal application did not (Fig. 9). Note, V_M_ measures were conducted in the presence of Y-27,632 to ensure that constriction didn’t introduce a motion artifact into electrical recordings^27^. Furthermore, measures of [Ca^2+^]_i_ supported the previous work in showing that superfused agonist (phenylephrine or U46619) elicited a rise in smooth muscle that was diltiazem (L-type Ca^2+^ channel blocker) sensitive. In contrast, the [Ca^2+^]_i_ response to focal agonist application was markedly muted and resistant to diltiazem, indicating a shift towards pharmacomechanical coupling and the use of a voltage-insensitive Ca^2+^ source (Fig. 10).

## Discussion

This study delved into receptor mediated constriction and the interplay among electro- and pharmacomechanical coupling mechanisms^25^. Work began by constructing concentration response curves to classic G_q/11_ (phenylephrine) or G_q/11_-G_12/13_ (U46619) agonists with and without an L-type Ca^2+^ channel blocker to separate among the coupling processes. Nifedipine diminished and rightward-shifted the concentration-response curves, consistent with electromechanical preceding pharmacomechanical responses, the so-called functional coupling bias. Further analysis of pharmacomechanical coupling revealed that phenylephrine phosphorylated CPI-17, an upstream regulator target of PP1c (MLCP catalytic subunit) where U46619 additionally phosphorylated MYPT1 (MLCP targeting subunit). Complementary tone measures aligned with the biochemistry data, with PKC blockade abolishing the pharmacomechanical response to phenylephrine while that induced by U46619 required both PKC and Rho-kinase inhibition. Subsequent work revealed that the order and dominance of the coupling mechanism “switched” if the number of cells stimulated was restricted though focal rather than global agent application. Clearly then, the coupling mechanisms underlying agonist-induced tone aren’t set in strict relative proportion to one another. Rather, dependent upon the concentration and manner of presentation, agonists trigger a range of vasomotor signatures, functionally biased toward electromechanical or pharmacomechanical coupling. We posit this flexible design is necessary for vascular networks to match blood flow delivery among tissue regions, whose metabolic requirements are disparate and variable with time or other physiological input.

### Functional bias in vascular smooth muscle

Arterial networks are responsible for setting how much and where blood flow is delivered within an organ^1,2^. In peripheral tissues like the gut, arterial tone is defined by the balance between constrictor and dilatory stimuli, the former in response to pressure, sympathetic transmitter release, and paracrine agents derived from endothelium and platelets. Each stimuli works through a G-protein coupled receptor, the resultant constriction dependent on two general mechanisms, an electromechanical process linked to V_M_ and Ca^2+^ influx through L-type Ca^2+^ channels, and pharmacomechanical coupling, a voltage-insensitive pathway tied to MLCP regulation^26^. While studies have long noted that agonists activate both mechanisms, what’s unclear is whether their relative contribution is fixed^5^ or variable, the latter enabled in part the receptor signaling bias that arises as ligand occupancy^17,27^, receptor density^28^ or structural configuration changes^29,30^. It’s in this context that we examined mesenteric contractile responses to two standard agonists, in the absence and presence of nifedipine, an L-type Ca^2+^ channel blocker that separates electro- from pharmacomechanical coupling^31^. The agonists of interest are phenylephrine and U46619, stable analogs of agents released from perivascular nerves and endothelium or platelets, respectively^32,33^. Phenylephrine confirmed through use of YM254890 to mediate constriction through G_q/11_ coupled α1-adrenoceptors, and U46619 via TXA_2_ receptors coupled to G_q/11_ & G_12/13_ (Fig. 2)^34^. Irrespective of the agonist and the G-protein coupling, nifedipine attenuated constriction and induced a rightward shift in the concentration response curve (Fig. 3). This shift is consistent with a functional signaling bias, with electromechanical coupling preceding but giving way to pharmacomechanical coupling as noted in Fig. 3A-B, right. Thus in contrast to coupling mechanisms presumptively activated in fixed relative proportion, our work indicates they are engaged in a defined sequential order^31^. Ensuing analysis also revealed that phenylephrine induced quicker temporal engagement of electromechanical coupling than pharmacomechanical (Fig. 3C and D). A similar trend wasn’t observed with U46619, a difference difficult to resolve but likely tied to the TXA_2_ receptor’s more complex G-protein coupling arrangement. Note, control experiments confirmed that qualitatively similar findings were obtained in mesenteric arteries lacking Ca_V_3.1, a T-type Ca^2+^ channel with a limited role in tone development (Fig. 4)^21^.

## Foundation of pharmacomechanical coupling

While electromechanical coupling, with its linkage to blood flow regulation, has been studied in depth, pharmacomechanical coupling has received decidedly less attention due to noted difficulties in conducting classic western blot analysis on small resistance vessels^12,35^. Classic literature typically ties pharmacomechanical coupling to the regulation of MLCP through its (1) catalytic subunit (PP1c) via CPI-17 phosphorylation and (2) targeting subunit (MYPT1) via T-855 and T-697 phosphorylation (Fig. 5A)^36^. In line with this perspective, we performed an amplified three-step western blot approach on mouse mesenteric arteries, with phenylephrine and U46619 application notable for elevating CPI-17 phosphorylation (Fig. 5) through PKC activation^25^. PKC is a classic downstream target of G_q_ coupled receptors activated through phospholipase C and the production of diacylglycerol and IP_3_; the latter drives Ca^2+^ release from the sarcoplasmic reticulum^37^. Interestingly, subsequent work revealed that while phenylephrine had no effect on MYPT1, U46619 enhanced phosphorylation at sites T-855 and T-697, which are under Rho-kinase control. Rho-kinase is a downstream target of G_12/13_ coupled receptors activated by RhoA, a small GTPase under the regulatory control of guanine exchange factors^38^. Consistent with these biochemical observations, functional work reveals that Calphostin C, a broad-spectrum PKC antagonist eliminated the ability of phenylephrine to induce nifedipine-insensitive constriction (Fig. 6). In contrast, PKC and Rho-kinase inhibition were both required to abolish the nifedipine-insensitive constriction by U46619, an agonist that mediates its effects through TXA_2_ receptors that are G_q/11_ and G_12/13_ coupled (Fig. 2B)^39^. The variability in the phosphorylation pattern highlights the interplay amongst agonists, signaling pathways and the molecular underpinnings of pharmacomechanical coupling. Final note, while MYTP1 is a recognized Rho-kinase target, its effects on pharmacomechanical coupling may also involve the modulation of actin stress fibers, a worthy subject for future study^40–42^.

## Altering functional bias of resistance arteries

Our initial work showed that coupling mechanisms are sequentially, rather than concurrently activated, with electromechanical dominating at low agonist concentrations and pharmacomechanical activating in accordance with dosage. A query that logically follows is whether the functional bias towards electromechanical coupling, presumptively enabled receptor signaling bias, can switch to full pharmacomechanical dominance. Consider in detail the computer model in Fig. 7, in which a small depolarizing current is injected into a variable number of smooth muscle cells within the virtual artery. When all are stimulated, we observed a pronounced depolarization of 12.5 mV, a response that starkly contrasts the < 2.0 mV depolarization noted when the number of stimulated smooth muscle cells is reduced to 1/8 of total. The latter finding is an intriguing but predictable change that arises when charge from a small number of stimulated cells disperses within the greater mass of unstimulated cells. It suggests that pharmacomechanical coupling is likely the dominant contractile mechanism in scenarios where agents are presented focally and discretely. This conceptual insight aligns well with experiments showing that focal agonist application elicits discrete nifedipine-insensitive constrictions (Fig. 8). PKC is prominent in mediating these discrete events, as responses were markedly attenuated following calphostin C application. We further confirmed the absence of arterial depolarization to focal agonist application (Fig. 9) while global superfusion-induced robust depolarization (~ 10–12 mV). This shift to pharmacomechanical dominance was also evident in our [Ca^2+^]_i_ measures; focal application elicited a modest, nifedipine-insensitive rise whereas the large response to global application was abolished by this L-type Ca^2+^ channel blocker (Fig. 10).

In considering the preceding work on MLCP regulation and pharmacomechanical coupling, it’s important to not overlook the necessity for a modest, voltage-insensitive Ca^2+^ source to maintain limited MLCK activity. While its foundation is unclear, it could be linked to Ca^2+^ waves, asynchronous events in vascular smooth muscle that spread end-to-end in a slow (100–3000 ms), repetitive manner^43,44^. Agonist-induced Ca^2+^ waves are insensitive to voltage, arise in response to IP3 receptor activation, and are associated with arterial constriction in preparations including mesentery^45^. This implies that IP3 receptor-mediated Ca^2+^ release plays a critical role in voltage-independent contraction, a key feature of pharmacomechanical coupling. Although not probed in detail, note that 2-APB, an agent with interferes with IP3 receptors and SR-Ca^2+^ mobilization, reduced the focal, voltage-independent constriction initiated by phenylephrine or U46619 (Fig. 8). A more thorough analysis awaits, one including measures of Ca^2+^ wave in arteries (1) treated with pharmacological agents that target key integral membrane proteins or (2) genetically modified to alter Ca^2+^ release/uptake from the sarcoplasmic reticulum^46,47^.

### Perspectives and significance

The foundation of excitation-contraction coupling is decidedly more complex for vascular tissue than for cardiac or skeletal muscle. In addition to membrane potential driving Ca^2+^ influx (electromechanical), a nonelectrical (pharmacomechanical) component tied to MLCP regulation must be carefully weighted and assigned a physiological role. The latter is difficult to ascertain if one adopts the traditional view that coupling mechanisms are set in fixed relative proportion, as both would presumptively support the same contractile behaviours^48^. Greater clarity, however, emerges as one considers functional bias and the ability of agonists to elicit a diversity of vasomotor “signatures”. First consider vasoactive agents circulating in blood or released from perivascular nerves at low concentration, a scenario where the ensuing vasomotor responses would be biased towards electromechanical coupling. The charge generated in support of depolarization would readily integrate among connected cells, across multiple arterial segments, a scenario ideal for setting base blood flow magnitude across a network. With stronger stimulation and further elevation of concentration, electrical control would cede to pharmacomechanical coupling, with a portion of tone development being resistant to arterial V_M_ modulation. In this scenario, hyperpolarizing responses, due to feedback loops or endothelial activation, would lose their ability to fully dilate an artery. Moderation of pharmacomechanical control would require an alternative nonelectrical mechanism, perhaps one tied to nitric oxide and protein kinase G, which could conceivably limit MLCP inhibition by interfering with MYPT1 phosphorylation^48–50^. Note, if agent concentration was high and focal enough, the result of local varicosity release, intrinsic tissue production or injury, small arterial segments could operate independent of the broader network, to tune blood flow distribution discretely and markedly to defined regions^51^. The idea of local pharmacomechanical control should be carefully weighed in context to arterial vasospasm, a disease state notably insensitive to dihydropyridines^52^. Compelling results in this study indicate a more effective means of ameliorating this deleterious state would be to therapeutically target signaling proteins (e.g. PKC or Rho-kinase) within non-electrical (i.e. pharmacomechanical) coupling pathways^53–55^.

## Conclusion

This study presents three key findings, the first being that constrictor agonists working through the α1-adenoreceptor or TXA_2_ receptors elicit functionally biased responses, with electromechanical preceding, but yielding to, pharmacomechanical coupling as concentration rises. Second, our work reveals a key role for PKC and Rho-kinase in pharmacomechanical coupling, the former enhancing CPI-17 phosphorylation (regulator of the catalytic subunit PP1c), and the latter MYPT1 phosphorylation (the myosin phosphatase targeting subunit). Lastly, we demonstrate that the functional bias toward electromechanical coupling can switch to pharmacomechanical dominance by simply changing how agents are delivered/presented to arteries. We conclude that agonists elicit a dynamic range of vasomotor signatures, each presumptively important to the real time control of arterial networks and the setting of blood flow delivery. The concepts herein underpin a framework to explore: (1) a broader range of GPCR agonists (including dilators) and vascular beds; and (2) sex-specific differences^56,57^ as work was restricted to males to limit confounding variables. They also provide new insight on vascular disease and the pathobiological basis of drug resistance hypertension and arterial vasospasm.

### Methods

#### Animal and tissue preparation

Animal procedures were approved by the animal care committee at the University of Western Ontario, in accordance with guidelines set forth by the Canadian Council on Animal Care and ARRIVE guidelines. Male C57BL/6J mice (wild type, 16–20 weeks of age) obtained from Jackson Laboratories were euthanized by CO_2_ asphyxiation^58^. The mesentery carefully removed and placed in cold PBS solution (pH 7.4) containing (in mM): 138 NaCl, 3 KCl, 10 Na_2_HPO_4_, 2 NaH_2_PO_4_, 5 glucose, 0.1 CaCl_2_, and 0.1 MgSO_4_. Fourth order arteries were dissected free of connective tissue and cut into 2–3 mm segments for further experimentation^59^. Experiments were done with the EC intact as the EC is essential for proper integrated electromechanical control. Mesenteric arteries from male Ca_V_3.1^−/−^ mice (global knockout, in house colony; 16–20 weeks of age) were also used in one experimental subset; homozygotes were generated from Ca_V_3.1^−/−^ mice crossed onto a C57BL/6J background^60^.

### Vessel myography

Isolated mesenteric arteries were placed in a pressure myograph system, cannulated, and equilibrated (intravascular pressure, 15 mmHg for 20 min) with warm (37ºC) physiological saline solution (PSS; 5% CO2, balance air) containing (in mM): 119 NaCl, 4.7 KCl, 1.7 KH_2_PO_4_, 1.2 MgSO_4_, 1.6 CaCl_2_, 10 glucose, and 20 NaHCO_3_. Vessel reactivity was then assessed by applying 60 mM KCl to the bath and measuring the diameter via an automated edge detection system (IonOptix, MA) and a 10x objective. Following wash off in standard PSS, intravascular pressure was incrementally increased to 60 mmHg and two sets of experiments were performed. First, we assessed the responsiveness of mesenteric arteries to superfused phenylephrine (3 × 10^− 9^ to 10^− 4^ M) or U46619 (10^− 9^ to 10 ^− 6^ M) in the absence and presence of YM254890 (0.1 µM, G_q−11_ inhibitor), nifedipine (0.3 µM, L-type Ca^2+^ channel blocker), calphostin C (0.3 µM, PKC inhibitor), or Y-27,632 (20 µM, Rho-kinase inhibitor). The percent maximal constriction was calculated as [*100×(D0–D)/D0-Dm*], where *D0* is diameter at 60 mmHg (no agonist), *D* is the diameter at each agonist concentration, and the *Dm* is the maximal constriction at the highest agonist concentration under control conditions. Constriction rate is calculated as [Δ diameter in response to agonist / time to max constriction]. In the second experimental set, phenylephrine (100 µM) and U46619 (10 µM) were focally applied upstream through small-bore glass micropipettes (1–2 μm tip, pressure ejection (30 psi, 10s pulse)), in the absence and presence of nifedipine (0.3 µM), calphostin C (0.3 µM) or 2-APB (50 µM); diameter was measured proximal to and distal from the site of agonist application.

### Arterial membrane potential (V_M_)

Arterial V_M_ was assessed by inserting a glass microelectrode backfilled with 1 M KCl (tip resistance = 90–110 MΩ) into the arterial wall^61^. V_M_ was first assessed under control conditions and following global application of phenylephrine (30 µM) or U46619 (0.1 µM) in the superfusate. In a second set of experiments, V_M_ was measured prior to and following the focal stimulation by phenylephrine (100 µM, 10s pulses) or U46619 (10 µM, 10s pulses). Criteria for a successful recording are: (1) sharp negative V_M_ deflection on electrode insertion; (2) stable V_M_ reading for a minimum of 2 min after entry; and (3) sharp return to baseline on electrode removal. To prevent vessels from overly constricting and introducing a motion artifact, 20 µM Y-27632 was present in the superfusate^62^).

### Intracellular Ca^2+^ ([Ca^2+^]_i_) measurements

To evaluate [Ca^2+^]_i_, mouse mesenteric arteries were isolated and loaded with 70 µM Fura-2-AM (dissolved and diluted in 50 µl DMSO, 3.4 µl Pluronic acid and 955 µl HBSS buffer) for 1 h at room temperature in the dark^63^. Arteries were then mounted in a pressure myograph and placed on the stage of an inverted epifluorescence microscope as per the vessel myography procedure to the point where vessel viability was verified with 60 mM K^+^. Fura-2 was alternatingly excited at 340 nm and 380 nm (5 Hz, 200 ms alternating intervals); emission spectra (510 nm) was collected on a RETRA light engine camera (Lumencor) viewed through a 10X objective (1.2 NA). Data was analysed using Nikon NIS Elements (AR 4.20.01) software. Background fluorescence was subtracted and the ratio of emitted fluorescence (F_340_/F_380_) calculated as a measure of [Ca^2+^]_i_. Experimentally, [Ca^2+^]_i_ was continuously monitored while vessels were treated with phenylephrine (100 µM focal or 30 µM global) or U46619 (10 µM focal or 0.1 µM global) in the presence or absence of diltiazem (30 µM, an L-type Ca^2+^ channel blocker with reduced photosensitivity). [Ca^2+^]_i_ was quantitated at baseline (5 s average) and once vasomotor tone had peaked to agonist application (5 s average; 70 and 10 s after global and focal treatment, respectively) Three regions of interest (1 × 1 μm) were defined within the analysis software and positioned on the vessel wall where it was sharply focused. For focal application of agonists, regions of interest were placed at the site of focal stimulation. 6 arteries from 6 mice were used for each experiment.

### Western blot analysis

A three-step western blotting protocol was used to quantify the phosphorylation state of MYPT1 (T-855 or T-697) and CPI-17 to agonist application^64^. Mesenteric arteries were dissected and cut in half (~ 3 mm in length), with two segments placed in a small silicone-covered dish containing Ca²⁺ PSS (37 °C, 15 min). After equilibration, one segment was left untreated, and the other was exposed to phenylephrine (30 µM) or U46619 (0.1 µM) for 3 min. Arteries were then transferred to a dish containing buffered acetone (10% trichloroacetic acid plus 10 mM dithiothreitol in acetone) to halt protease and phosphatase activity. This fixation step was followed by overnight freeze-drying (VirTis Bench Top 3.5 Pro, -55 °C). Freeze dried samples were then transferred to sample buffer (75 mM Tris HCL (pH 6.8), 10% Glycerol, 6% SDS, Bromophenol Blue, 2-mercaptoethanol) and vortexed overnight. Proteins were then separated on a 4–10% gradient SDS-PAGE or a phosphate-affinity tag SDS-PAGE (for CPI-17), and then transferred to nitrocellulose^65^. Membranes were blocked for 1 h (5% nonfat dairy milk in Tris-buffered saline), and then incubated with a primary antibody (p-MYPT1-T697, p-MYPT1-T855, CPI-17; 1:500) followed by a biotin-conjugated secondary antibody (1:5,000) and HRP-conjugated streptavidin (1:10,000). The membrane was washed repeatedly with Tris Buffered Saline with Tween (TBST) after each incubation step. The blot was developed using Amersham ECL Prime Western Blotting Reagent and imaged on a Gel Doc using Image Lab software (Bio-Rad). The density of each band was quantified using scanning densitometry^65^. MYPT1 phosphorylation was normalized to α-smooth muscle actin; phosphorylated CPI-17 was normalized to total CPI-17 expression. Agonist treated vessels were then normalized in relation to untreated vessels.

### Computational modeling

#### Construction of virtual artery

A 500 μm bilayer artery model with an inner endothelial cell (EC) layer and a surrounding smooth muscle cell (SMC) layer was developed (refer to Fig. 7A)^14,66^. Cells were electrically coupled with gap junctional resistance set at 3 MΩ (EC-to-EC), 90 MΩ (SMC-SMC), and 1800 MΩ (EC-SMC; note each smooth muscle cell was linked to two ECs. Reaction currents simulated ion channel activity; arterial diameter was initially set at 75 μm, and was adjusted to SMC membrane potential changes, as modeled through ordinary differential Eqs^14,67^. Simulations consisted of injecting a variable number of smooth muscle cells (All, 1/2, 1/4, 1/8) with a current of + 0.83 pA. The depolarizing current when applied to all smooth muscle cells elicited a + 12 mV depolarization, a response in line with physiological observations^68^. Greater detail as to model construction can be found in the original publication^14^.

### Numerical methods

A Mersenne Twister algorithm facilitated random EC-SMC coupling, with cell electrical activity computed by our ordinary differential equations using a high-order implicit backward difference formula^69,70^. Distributed memory parallelization was implemented using the MPI library^71^. The simulations were advanced over several iterations, until the relative solution changes over consecutive iterations were negligible (i.e., relative tolerance less than 10^− 6^) at which point steady state was assumed. The simulations were performed on a local HPC cluster running Fedora 31.

### Statistical analysis

Data are expressed as mean ± SD and n indicates the number of samples or arteries. Power analysis was performed a priori to assess the sample size sufficient for obtaining statistical significance. Using preliminary/published data, we predict an *n* = 6 and *n* = 4 animals sufficiently powers (*p* = 0.80, a = 0.05) statistical analysis for myography and western blot experiments, respectively^24^. No more than 1 experiment was performed on cells/tissues from any given animal. Nonlinear curve fitting method of GraphPad Prism 10.1 software was used to analyze the accumulative dose- effect curves. Two-way ANOVA and paired *t* tests were performed to compare the effect of a given condition/treatment on diameter, V_M_, intracellular [Ca^2+^], or phosphorylation state. *P* values ≤ 0.05 were considered statistically significant.

#### Chemicals

Primary and secondary antibodies were obtained from the following sources: Anti-Phospho-MYPT1 (Thr697) and (Thr855) rabbit polyclonal antibody was purchased from New England Labs. Anti-α smooth muscle actin rabbit polyclonal antibody was purchased from Abcam. Anti-CPI-17 antibody rabbit polyclonal IgG, and Biotin-sp conjugate goat anti-rabbit IgG antibody Millipore Sigma. Y-27,632 and U46619 were purchased from Tocris. YM254890 was acquired from Focus Biomolecules and calphostin C was acquired from Cayman Chemical. Nifedipine, phenylephrine hydrocholoride, diltiazem hydrocholoride, Streptavidin-peroxidase and Fura-2 LR/AM and all other chemicals were obtained from Millipore Sigma, unless stated otherwise. Where DMSO was used as a solvent, the maximal DMSO concentration after application did not exceed 0.05%.

## Electronic supplementary material

Below is the link to the electronic supplementary material.

Supplementary Material 1

Supplementary Material 2

Supplementary Material 3

## Data Availability

All data that support the findings of this study are available from the lead contact, Nadia Haghbin (nhaghbin@uwo.ca), upon reasonable request. Original western blots are presented and concentration-response raw data points and are provided in separate files as supplementary dataset.
