# Supplementary material for: Functional bias of contractile control in mouse resistance arteries
Source: Sci Rep. 2024 Oct 22;14:24940. doi: 10.1038/s41598-024-75838-8 (PMC11496727; doi:10.1038/s41598-024-75838-8)

Figure 5B- Representative blots

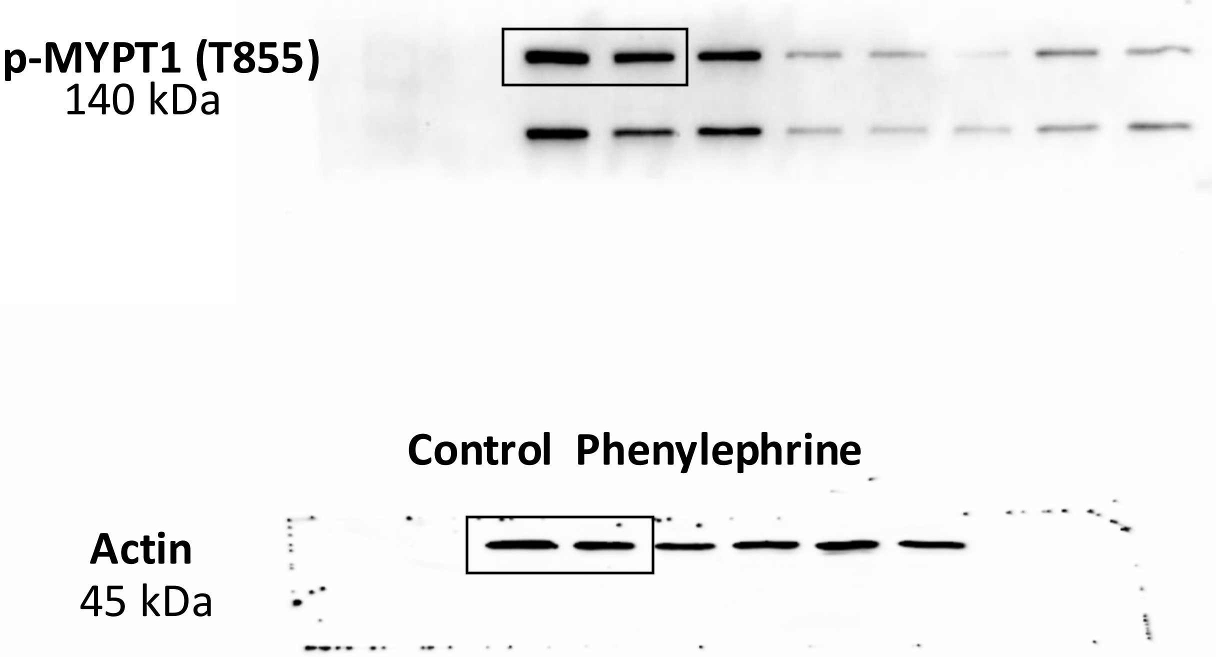

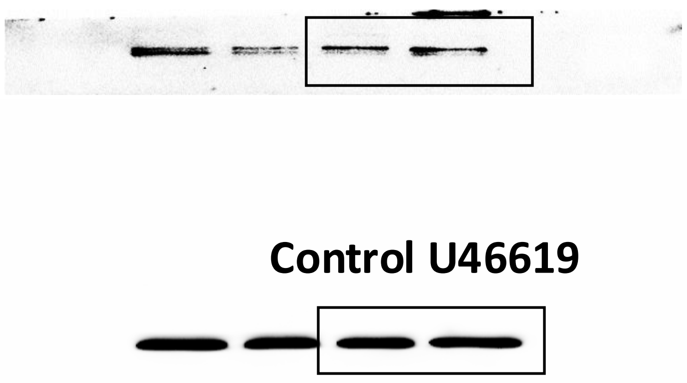

The grouping of blots cropped from different parts of the same gel.

Figure 5C- Representative blots

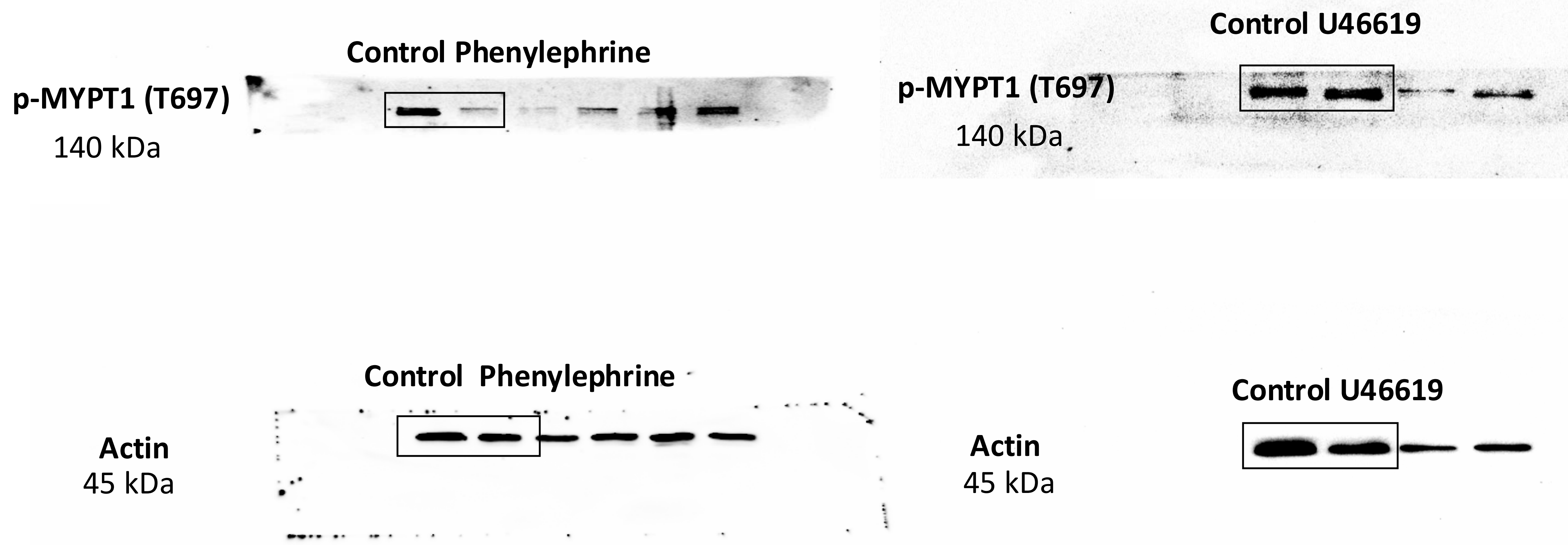

Figure 5D- Representative blots

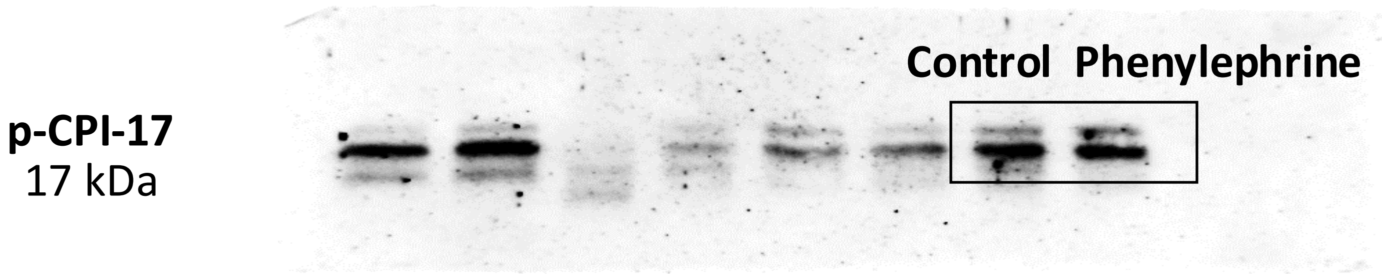

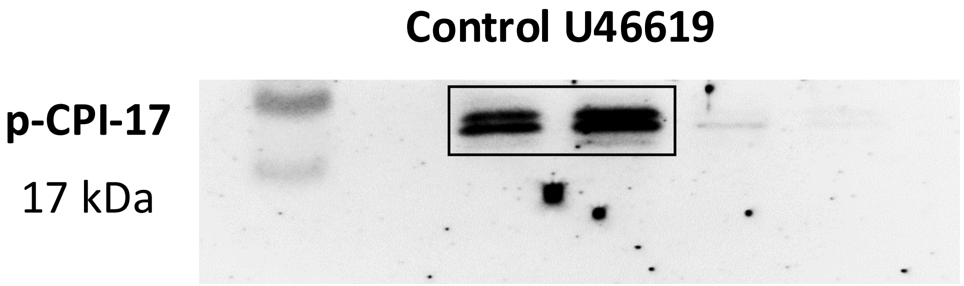

Supplement: Supplementary file 3 — Supplementary Material 3 [file 41598_2024_75838_MOESM3_ESM.pdf]
